# Radiomics-based infarct features on CT predict hemorrhagic transformation in patients with acute ischemic stroke

**DOI:** 10.3389/fnins.2022.1002717

**Published:** 2022-09-21

**Authors:** Gang Xie, Ting Li, Yitao Ren, Danni Wang, Wuli Tang, Junlin Li, Kang Li

**Affiliations:** ^1^North Sichuan Medical College, Nanchong, China; ^2^Department of Radiology, Chongqing General Hospital, Chongqing, China

**Keywords:** acute ischemic stroke, hemorrhagic transformation, computed tomography, radiomics, prediction

## Abstract

**Objective:**

To develop and validate a model based on the radiomics features of the infarct areas on non-contrast-enhanced CT to predict hemorrhagic transformation (HT) in acute ischemic stroke.

**Materials and methods:**

A total of 118 patients diagnosed with acute ischemic stroke in two centers from January 2019 to February 2022 were included. The radiomics features of infarcted areas on non-contrast-enhanced CT were extracted using 3D-Slicer. A univariate analysis and the least absolute shrinkage and selection operator (LASSO) were used to select features, and the radiomics score (Rad-score) was then constructed. The predictive model of HT was constructed by analyzing the Rad-score and clinical and imaging features in the training cohort, and it was verified in the validation cohort. The model was evaluated with the receiver operating characteristic curve, calibration curve and decision curve, and the prediction performance of the model in different scenarios was further discussed hierarchically.

**Results:**

Of the 118 patients, 52 developed HT, including 21 cases of hemorrhagic infarct (HI) and 31 cases of parenchymal hematoma (PH). The Rad-score was constructed from five radiomics features and was the only independent predictor for HT. The predictive model was constructed from the Rad-score. The area under the curve (AUCs) of the model for predicting HT in the training and validation cohorts were 0.845 and 0.750, respectively. Calibration curve and decision curve analyses showed that the model performed well. Further analysis found that the model predicted HT for different infarct sizes or treatment methods in the training and validation cohorts with 78.3 and 71.4% accuracy, respectively. For all samples, the model predicted an AUC of 0.754 for HT in patients within 4.5 h since stroke onset, and predicted an AUC of 0.648 for PH.

**Conclusion:**

This model, which was based on CT radiomics features, could help to predict HT in the setting of acute ischemic stroke for any infarct size and provide guiding suggestions for clinical treatment and prognosis evaluation.

## Introduction

Hemorrhagic transformation (HT) is an important complication of acute ischemic stroke (AIS). According to the European Cooperative Acute Stroke Study II (ECASS II), HT is divided into four types (Hacke et al., 1998). Hemorrhagic infarct 1 (HI1) is defined as small spot hemorrhage along the infarct edge, hemorrhagic infarct 2 (HI2) as patchy or multiple confluent spotting hemorrhage in the infarct area with no space-occupying effect, parenchymal hematoma 1 (PH1) as hematoma < 30% of the infarct size with a slight space-occupying effect, and parenchymal hematoma 2 (PH2) as hematoma > 30% of the infarct size with a significant space-occupying effect. HT is closely related to the poor prognosis of patients. Previous studies have associated parenchymal hematoma with functional deterioration, but some studies found that small spot hemorrhage still affects long-term functional outcomes (van Kranendonk et al., 2019; Bivard et al., 2020). In addition, HT is also an important indicator for clinical treatment. When the risk of HT, especially symptomatic intracranial hemorrhage, is high, the advantages and disadvantages of intravenous thrombolysis (IVT) or mechanical thrombectomy (MT) need to be carefully evaluated (Hacke et al., 2008). Therefore, predicting HT at an early stage is of great importance. Previous studies have shown a variety of HT prediction methods based on clinical biological indicators, radiology or deep learning, all of which exhibited good prediction performance (Whiteley et al., 2012; Hao et al., 2017; Okazaki et al., 2017; Jiang et al., 2021). In recent years, radiomics has played an important role in the diagnosis, treatment, and prognosis of diseases. Radiomics features can effectively reflect the heterogeneity of lesions, and this quantitative index of microscopic differences provides a new method to analyze and understand diseases (Davnall et al., 2012). However, to our knowledge, only a few studies have predicted HT based on the radiomics features of magnetic resonance imaging and have shown promising results in this regard (Kassner et al., 2009; Zhai et al., 2022). To further explore the utility of radiomics features in predicting HT, this study aimed to construct a model based on the radiomics features of the infarct areas on non-contrast-enhanced CT in patients with AIS to evaluate its predictive value for HT before treatment.

## Materials and methods

### Patients

This retrospective study was approved by the medical ethics committee of the North Sichuan Medical College (No. [2022] 27), and the requirement for written informed consent was waived.

**FIGURE 1:**
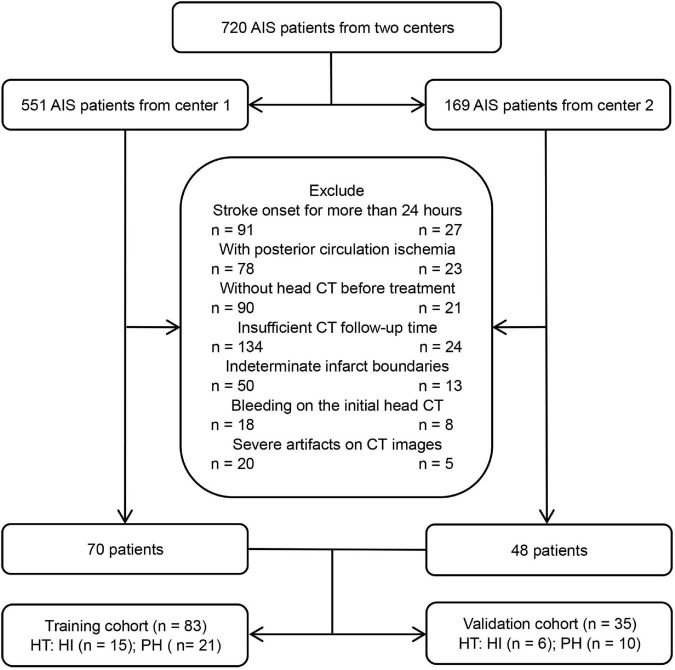
The flow chart of patient inclusion and exclusion criteria.

**FIGURE 2:**
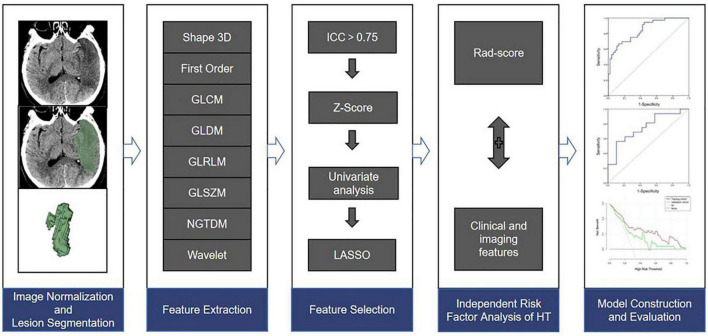
The flow chart of this study.

From January 2019 to February 2022, patients with AIS who were treated at the stroke center of the Affiliated Hospital of North Sichuan Medical College and Chongqing General Hospital were included. The inclusion criteria were as follows: (1) AIS patients with anterior circulation involvement who met the WHO diagnostic criteria (Aho et al., 1980), (2) the initial non-contrast-enhanced head CT data were collected before treatment within 24 h of symptom onset, (3) the follow-up time of head CT after treatment was not less than 7 days, unless HT occurred within 7 days, and (4) the boundary of the infarct areas could be determined by initial non-contrast-enhanced CT. The exclusion criteria were as follows: (1) bleeding on the initial head CT scan, (2) severe artifacts on CT images, and (3) incomplete data. Finally, 118 patients with AIS who met the criteria were included, and all patients were treated in strict accordance with the AIS guidelines (Powers et al., 2018). The flow chart of this study is shown in Figures 1, 2.

### Data collection

Data, such as age, sex, stroke onset time, National Institutes of Health Stroke Scale (NIHSS) score, and treatment methods, were collected. The patients’ smoking status and drinking habits as well as the presence of hypertension, diabetes mellitus, hyperlipidemia, atrial fibrillation, heart failure, coagulation disorder, dense middle cerebral artery sign, and massive cerebral stroke were assessed. Relevant information was obtained from the electronic medical record system and the picture archiving and communication system (PACS).

### Image acquisition and analysis

All patients’ head non-contrast-enhanced CT images were acquired by LightSpeed VCT (GE) or IQon Spectral CT (Philips) scanning. The patients were placed in the supine position, and the orbitomeatal line was used as the scanning baseline. The scanning range was from the top of the head to the base of the skull. The scanning parameters were as follows: tube voltage 120 kV, tube current 220 mAs, pitch 1.0, layer thickness and layer spacing 5 mm.

Two radiologists with more than 5 years of experience analyzed the patient’s CT images independently: (1) the boundary of the infarct areas was defined by marked low-density areas in the initial CT after adjusting the gray value of images, (2) the dense middle cerebral artery sign was defined as a higher density of the middle cerebral artery on the infarcted side than on the contralateral side, (3) massive cerebral stroke was defined as the infarct areas exceeding 1/3 of the cerebral hemisphere, and (4) HT was defined as a lack of high-density shadow (CT attenuation values were approximately 60–90 Hu) in or around the infarcted areas on the initial CT when high-density shadows appeared on the follow-up CT and persisted for ≥2 days or when a high-density shadow persisted after iodine removal by the virtual non-contrast (VNC) of IQon spectral CT. Kappa analysis was used to evaluate the consistency of the diagnosis of two radiologists, with details described in the Supplementary Table 1. Differences in the results were discussed and eventually agreed upon.

### Infarct lesion segmentation, feature extraction, and selection

The 3D-Slicer^1^ was used for infarct lesion segmentation and feature extraction. The initial non-contrast-enhanced head CT images of all AIS patients were imported into the software in DICOM format for analysis.

The specific process was as follows: (1) For image normalization and lesion segmentation, the boundary of the infarct areas was determined by adjusting the gray value of the CT images, and semiautomatic segmentation was then used to obtain the three-dimensional ROI of the infarct areas. All ROIs of images were normalized, including resampling the image voxels to 1 mm × 1 mm × 1 mm by linear interpolation, smoothing the images with a Gaussian filter, and fixing the bin width value of the image gray value at 25. (2) For radiomics feature extraction, the radiomics features were extracted from the ROIs of images using the Pyradiomics plugin in the software, including 3D-shaped features, first-order features, gray-level cooccurrence matrix (GLCM), gray-level dependence matrix (GLDM), gray-level run length matrix (GLRLM), gray-level size zone matrix (GLSZM), neighboring gray-tone difference matrix (NGTDM), and wavelet-based features. (3) For radiomics feature selection, and to ensure stability, 60 AIS patients were randomly selected by the same radiologist one week later for repeated ROI segmentation and feature extraction, and the intraclass correlation coefficient (ICC) was then used to evaluate the consistency of the two features. Features with ICC > 0.75 were normalized with the *Z* Score. Feature selection was necessary to avoid overfitting. Firstly, univariate analysis (Student’s *t*-test or Mann–Whitney *U* test) was used to select features with differences between groups, and the least absolute shrinkage and selection operator (LASSO) and 10-fold cross validation were then used to determine the optimal texture features related to HT. Finally, the feature equation was established via logistic regression. The radiomics score (Rad-score) for each patient was the sum of the products of the regression coefficients and the features in the equation.

### Model construction and evaluation

All patients were randomly divided into a training cohort (83 patients) and a validation cohort (35 patients) according to the principle of stratified randomization at a ratio of 7:3. In the training cohort, the Rad-score and clinical and imaging features of patients were subjected to a univariate analysis, and variables with *P* < 0.05 in the univariate analysis were included for multivariate analyses. Variables with *P* < 0.05 in multivariate analyses were determined to be independent risk factors for HT, and logistic regression was then used to construct a model. Moreover, the model was validated in the validation cohort, and the prediction performance of the model in different scenarios was further discussed hierarchically. A receiver operating characteristic curve analysis was used to calculate the area under the curve (AUC) of the model, a calibration curve was used to evaluate the consistency between the predicted probability and the actual probability of the model, and clinical decision curve analysis was used to evaluate the clinical net benefit of the model.

### Statistical analysis

A statistical analysis was performed using SPSS (version 26.0, IBM, Armonk, NY, USA) and R software (version 4.1.0).^2^ Continuous variables with a normal distribution were described as the mean ± standard deviation (SD), continuous variables with a skewed distribution were described as the median [interquartile range (IQR)], and categorical variables were described as the frequency and constituent ratio (%). In the univariate analysis, Student’s *t*-test or the Mann–Whitney *U* test was used for continuous variables, and the chi-squared test or Fisher’s exact test was used for categorical variables. Logistic regression was used for multivariate analysis. A two-sided *P* < 0.05 was considered statistically significant.

## Results

### Radiomics score development

**FIGURE 3:**
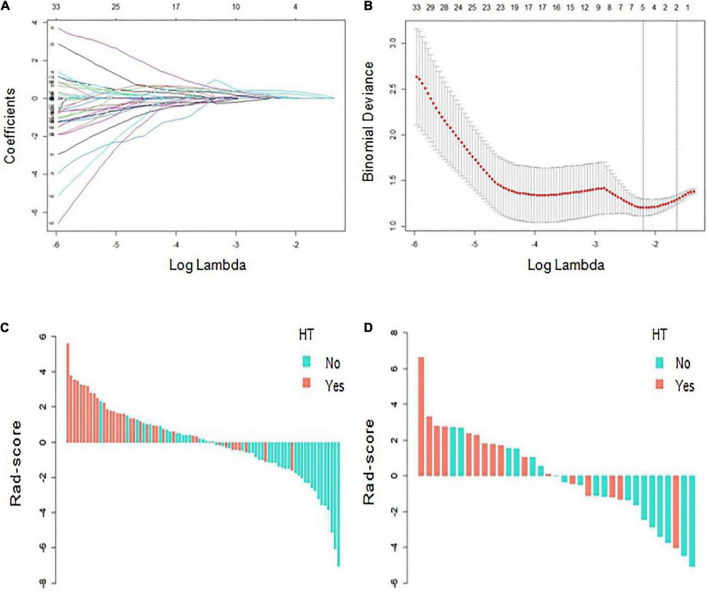
Radiomics score (Rad-score) development. **(A,B)** Radiomics feature selection based on least absolute shrinkage and selection operator (LASSO) and 10-fold cross-validation. When binomial deviance is the smallest, a total of five optimal features are obtained. **(C,D)** The Rad-score of each patient in the training and verification cohorts. Red indicates hemorrhagic transformation (HT), and blue indicates non-HT.

Based on the ROI of the infarcted areas on non-contrast-enhanced CT images, a total of 851 radiomic features were extracted from each patient. After ICC and univariate analyses, 201 stable features with differences between groups were screened out, with details described in the Supplementary. Finally, five features that are highly related to HT were selected by LASSO and 10-fold cross validation (Figures 3A,B). The five features were all wavelet-based features, including one wavelet-filtered GLCM feature and two wavelet-filtered GLDM and GLRLM features each. Logistic regression was used to analyze the five features and establish an equation, and the Rad-score was calculated according to the regression coefficients in the equation. Rad-score = 1.022 × (wavelet-LLH-InverseVariance) + 0.996 × (wavelet-LLL-DependenceNonUniformity) + 0.978 × (wavelet-LHL-RunVariance) - 0.177 × (wavelet-LHH-DependenceNonUniformityNormalized) - 0.004 × (wavelet-LLH-RunLengthNonUniformity). The Rad-scores for each patient in the training and validation cohorts were used to generate a waterfall plot (Figures 3C,D).

### Baseline characteristics and evaluation of independent risk factors for hemorrhagic transformation

Of the 118 patients with AIS, 63 were males and 55 were females, and the patients were aged from 42–94 (69.22 ± 12.33) years. Ultimately, 52 patients developed HT and 66 did not. Among them, 21 cases were HI and 31 cases were PH. The baseline characteristics of patients with HT and without HT in the training and validation cohorts are compared in Table 1. There were statistically significant differences in variables such as massive cerebral stroke, NIHSS score and Rad-score between the HT and non-HT groups (*P* < 0.05). In the training cohort, a multivariate analysis showed that only the Rad-score was an independent predictor for HT (Table 2). Moreover, the Rad-score of HT patients were higher than those of patients without HT (*P* < 0.05).

**TABLE 1:** Baseline characteristics of the hemorrhagic transformation (HT) and non-HT groups in the training and validation cohorts.

	Training cohort	(*n* = 83)		Validation cohort	(*n* = 35)	
						
Variable	HT (36)	Non-HT (47)	*P*-value	HT (16)	Non-HT (19)	*P*-value
Sex (male)	18 (21.69%)	29 (34.94%)	0.372	8 (22.86%)	8 (22.86%)	0.740
Age, year	69.14 ± 12.34	68.28 ± 12.08	0.750	67.56 ± 14.71	73.11 ± 10.88	0.210
Smoking	10 (12.05%)	14 (16.87%)	1.000	3 (8.57%)	6 (17.14%)	0.460
Habitual alcohol intake	10 (12.05%)	11 (13.25%)	0.800	4 (11.43%)	6 (17.14%)	0.723
Hypertension	26 (31.33%)	30 (36.14%)	0.483	12 (34.29%)	17 (48.57%)	0.379
Diabetes mellitus	9 (10.84%)	10 (12.05%)	0.794	2 (5.71%)	7 (20.00%)	0.135
Hyperlipidemia	18 (21.69%)	22 (26.51%)	0.827	8 (22.86%)	8 (22.86%)	0.740
Atrial fibrillation	20 (24.10%)	22 (26.51%)	0.509	9 (25.71%)	11 (31.43%)	1.000
Heart failure	20 (24.10%)	22 (26.51%)	0.509	9 (25.71%)	8 (22.86%)	0.505
Coagulation disorder	8 (9.64%)	6 (7.23%)	0.376	3 (8.57%)	0	0.086
Treatment methods			0.154			0.140
Non-reperfusion	22 (26.51%)	21 (25.30%)		9 (25.71%)	9 (25.71%)	
IVT	8 (9.64%)	12 (14.46%)		3 (8.57%)	3 (8.57%)	
MT	3 (3.61%)	12 (14.46%)		0	5 (14.29%)	
IVT with MT	3 (3.61%)	2 (2.41%)		4 (11.43%)	2 (5.71%)	
Dense MCA sign	14 (16.87%)	9 (10.84%)	0.053	5 (14.29%)	8 (22.86%)	0.727
Massive cerebral stroke	26 (31.33%)	12 (14.46%)	0.000	11 (31.43%)	5 (14.29%)	0.018
Stroke onset time, h	5.00 (3.00–10.00)	4.00 (2.00–9.25)	0.412	4.50 (2.00–10.00)	4.00 (3.00–5.00)	0.569
NIHSS score	15.97 ± 5.60	12.83 ± 6.02	0.017	17.63 ± 4.19	14.00 ± 3.59	0.009
Rad-score	1.39 ± 1.59	-1.07 ± 1.95	0.000	1.15 ± 2.46	-0.95 ± 2.32	0.014

Data are expressed as the mean ± standard deviation, median (interquartile range) or frequency (constituent ratio). IVT, intravenous thrombolysis; MT, mechanical thrombectomy; Dense MCA sign, dense middle cerebral artery sign; NIHSS score, National Institutes of Health stroke scale score; Rad-score, radiomics score.

**TABLE 2:** Multivariate logistic regression analysis of independent risk factors for hemorrhagic transformation (HT) in the training cohort.

Variable	Coefficient	OR (95% CI)	*P*-value
Massive cerebral stroke	0.01	1.01 (0.25–4.10)	0.994
NIHSS score	0.04	1.05 (0.95–1.16)	0.388
Rad-score	0.98	2.66 (1.47–4.82)	0.001

NIHSS score, National Institutes of Health Stroke scale score; Rad-score, radiomics score.

### Model construction and evaluation

**FIGURE 4:**
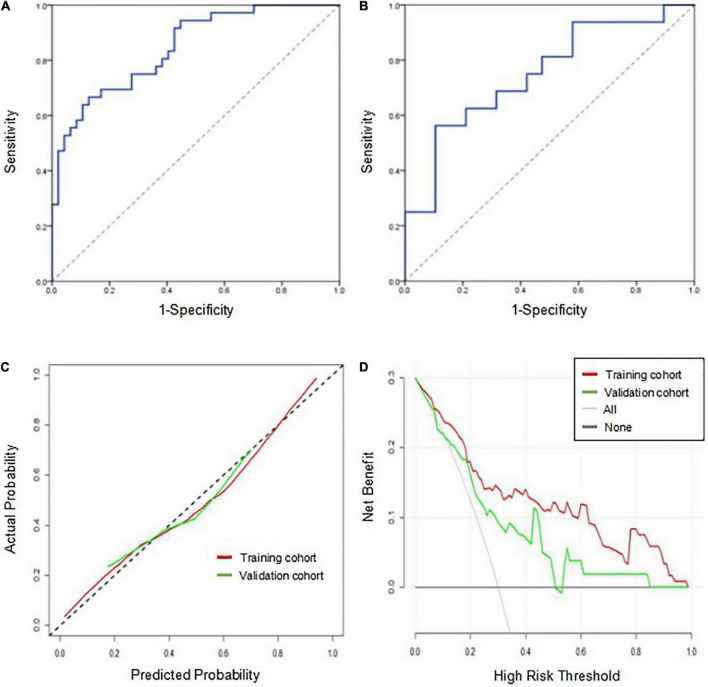
Performance evaluation of the model in the training and validation cohorts. **(A)** Receiver operating characteristic curve of the model in the training cohort. **(B)** Receiver operating characteristic curve of the model in the validation cohort. **(C)** The calibration curve of the model in the training and validation cohorts. The closer the calibration curve (solid line) is to the diagonal (dotted line), the higher the consistency between the predicted probability and the actual probability. **(D)** The clinical decision curve analysis of the model in the training and validation cohorts. When the red line and the green line are farther away from the white line on the right and the black line below, the net benefit is high.

**FIGURE 5:**
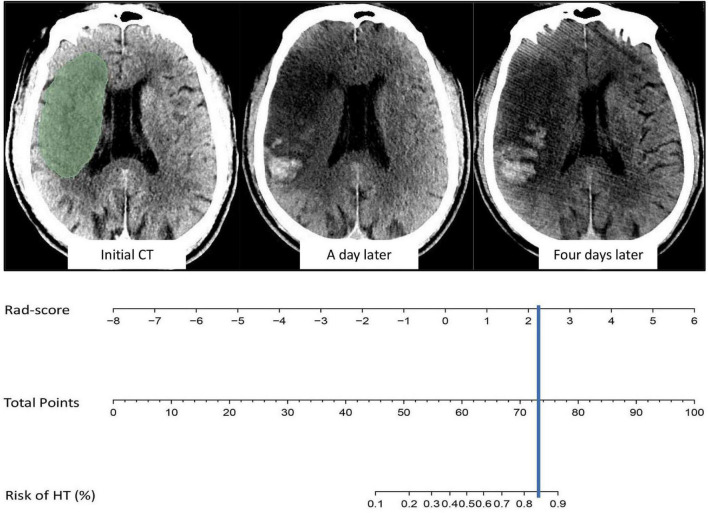
Example of model visualization prediction. The cerebral infarct area was segmented and radiomics features were extracted, and the value of Rad-score was then incorporated into the model for calculation. The results indicated an 80–90% probability of HT in the future, which was confirmed on head CT one and four days after intravenous thrombolysis.

The AUCs of the logistic regression model constructed based on the Rad-score for predicting HT in the training and validation cohorts were 0.845 (95% CI, 0.763–0.927) and 0.750 (95% CI, 0.585–0.915), respectively (Figures 4A,B). Moreover, the sensitivities in the training and validation cohorts were 0.667 and 0.562 and the specificities were 0.872 and 0.895, respectively. The calibration curve was highly consistent between the predicted and actual probabilities of the training and validation cohorts (Figure 4C). The clinical decision curve analysis showed that the model had a good net clinical benefit (Figure 4D). We visualized the model constructed from Rad-score, which intuitively predicted the risk of HT in AIS patients (Figure 5).

Although variables such as massive cerebral stroke and the NIHSS score were not independent predictors for HT in the multivariate analysis, they were related to HT (El Nawar et al., 2019). When the two variables were added to the model, the AUCs of the combined model for predicting HT in the training and validation cohorts were 0.849 (95% CI, 0.767–0.930) and 0.750 (95% CI, 0.586–0.914), respectively. The DeLong test showed that the combined model and the original model did not significantly differ (*P* > 0.05).

**FIGURE 6:**
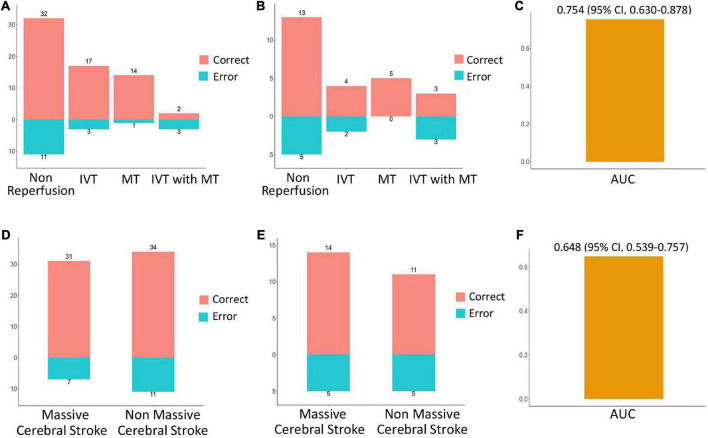
Performance evaluation of model in different scenarios. In the training cohort **(A)** and validation cohort **(B)**, the prediction accuracy of the model for HT in the setting of non-reperfusion therapy, intravenous thrombolysis (IVT), mechanical thrombectomy (MT), and IVT combined with MT therapy. In the training cohort **(D)** and the validation cohort **(E)**, the prediction accuracy of the model for HT in the setting of massive cerebral stroke and non-massive cerebral stroke. Red indicates a correct prediction, and blue indicates an incorrect prediction. For all samples, the prediction performance of the model for HT in patients within 4.5 h since stroke onset **(C)**, and prediction performance for parenchymal hematoma **(F)**.

The prediction performance of the model under different scenarios was further discussed hierarchically. For different treatment methods, the model predicted HT with 40.0–100.0% accuracy in the training and validation cohorts (Figures 6A,B). For massive cerebral stroke and non-massive cerebral stroke, the model predicted HT with 68.8–81.6% accuracy in the training and validation cohorts (Figures 6D,E). Overall, the model predicted HT in both settings with 78.3 and 71.4% accuracy in the training and validation cohorts, respectively. In addition, the model had no statistical difference in the prediction of HT for different treatment methods or different infarct size (*P* > 0.05). For all samples, the model predicted an AUC of 0.754 (95% CI, 0.630–0.878) for HT in patients within 4.5 h since stroke onset, and predicted an AUC of 0.648 (95% CI, 0.539–0.757) for PH (Figures 6C,F).

## Discussion

To our knowledge, this study was a new attempt to construct an HT prediction model based on the radiomics features of the infarct area on non-contrast-enhanced CT images. The model can assess the risk of HT after reperfusion or non-reperfusion therapy in AIS patients with anterior circulation involvement regardless of infarct size. The AUCs of the model in the training and validation cohorts were 0.845 and 0.750, respectively.

Radiomic features can quantitatively reflect the voxel differences in different image spaces, which represent the microscopic pathological changes and heterogeneity of lesions (Limkin et al., 2017; Guiot et al., 2022). Previous studies have shown that the occurrence of HT is mainly related to damage to the blood–brain barrier in the infarcted areas, reperfusion injury and coagulation disorders (Bai and Lyden, 2015; Yang et al., 2019; Zheng et al., 2019; Spronk et al., 2021). For AIS patients, the state of the blood–brain barrier in the cerebral infarction areas changes dynamically with the stroke onset time, the location and degree of thrombus, the infarct size and the body’s own physiological and pathological changes; consequently, different changes will appear on imaging (Wu et al., 2021). Compared with human visual analysis, radiomics features can more fully reflect the microscopic differences within the lesion and thus better evaluate the state of blood–brain barrier damage (Kassner et al., 2009; Valdés Hernández et al., 2017). In this study, a large number of radiomics features were extracted from the infarct areas of the non-contrast-enhanced head CT images of AIS patients, and five optimal features were finally obtained through selection. Among them, the two GLDM features reflect the similarity of image dependencies, the two GLRLM features reflect the similarity of image run lengths, and the GLCM features reflect the roughness of image texture. All features reflect the heterogeneity of the cerebral infarct area, that is, represent differences in the disruption of the blood-brain barrier. By comparing the Rad-score calculated from the five features, it was found that the Rad-scores of AIS patients who developed HT in the future were significantly higher than those of patients in the non-HT group in both the training and validation cohorts (*P* < 0.05).

Based on further analyses combined with clinical and imaging features, this study found that massive cerebral stroke and higher NIHSS and Rad-score increase the probability of future HT after stroke. Compared with smaller strokes, massive cerebral strokes are more often caused by severe vascular disease, such as internal carotid artery or middle cerebral artery embolism. The severe cytotoxic edema caused by ischemia and hypoxia in massive cerebral stroke significantly exacerbates the destruction of the blood–brain barrier, resulting in HT in the future (El Nawar et al., 2019; Muscari et al., 2020; Yoshimura et al., 2022). The NIHSS score is used to evaluate the degree of neurological deficit in AIS patients. In this study, the NIHSS score of HT patients was higher than that of patients without HT, with an average score of approximately 16–17. Other factors in the study did not significantly differ between the HT and non-HT groups, which is consistent with some previous reports but differs from the findings of other studies (Jickling et al., 2013; Jensen et al., 2020; Tian et al., 2022). This discrepancy may be related to differences in the data distribution and sample size of this study. In addition, only the Rad-score was an independent predictor of HT in the multivariate analysis of this study, suggesting that radiomic features may be more significant than clinical and imaging features in predicting HT.

To evaluate the performance of the model, we validated the constructed model and discussed the prediction accuracy of HT for different infarct sizes and different treatment methods. The results show that the model performed well in evaluating HT. In addition, further analysis showed that the model could still be used to predict HT in patients within 4.5 h since stroke onset, with an AUC of 0.754; it also could helped predict the occurrence of PH, with an AUC of 0.648. When the model predicts a higher risk of HT, irrespective of the occurrence of massive cerebral stroke, the treatment benefits for the patient needs to be comprehensively evaluated, especially regarding the selection of IVT or MT. Previous studies, such as those that developed the SPAN-100 and SICH models (Saposnik et al., 2013; Lokeskrawee et al., 2017), mainly focused on evaluating the occurrence of HT after IVT. Unlike the models described in these studies, the model established in our study is suitable for the evaluation of HT in the setting of various treatment methods, such as non-reperfusion therapy, IVT, MT, and IVT combined with MT. At present, very few studies have examined the prediction of HT by radiomics. A previous study performed a predictive analysis of HT based on the radiomic features of the infarct area in the postcontrast T1-weighted images of 34 AIS patients, with an AUC of 0.60–0.80 (Kassner et al., 2009). CT scanning is more convenient and rapid than magnetic resonance, and it is also the preferred imaging method for AIS patients within the time window. The applicability of the model can be expanded to a certain extent based on CT image analysis.

This study remains subject to some limitations. First, this study was retrospective and only examined a small sample, which may lead to biased results. Thus, the applicability of the model requires further prospective and multicenter validation with larger samples. Second, the image analysis suffered from errors in the segmentation of the infarct areas due to the limitation of the resolution of CT. Therefore, cerebral infarct volume on non-contrast-enhanced CT was not included in this study. To discuss the effect of infarct size on HT, we adopted the concept of massive cerebral stroke. Third, the model may no longer be applicable for some hyperacute AIS patients whose infarct boundary cannot be determined by adjusting the gray value of images. Further analysis based on cerebral perfusion imaging may provide more meaningful results.

In conclusion, we developed and validated a model based on the radiomics features of the non-contrast-enhanced head CT images of AIS patients. The model could help to evaluate the occurrence of HT before treatment to provide guidance for clinical treatment and prognosis evaluation.

## Data availability statement

The original contributions presented in this study are included in the article/Supplementary material, further inquiries can be directed to the corresponding author.

## Ethics statement

The studies involving human participants were reviewed and approved by the North Sichuan Medical College. Written informed consent for participation was not required for this study in accordance with the national legislation and the institutional requirements.

## Author contributions

KL and GX: design the experiments. TL, YR, and GX: acquisition of the data. WT, DW, and GX: analysis of the data. JL and KL: study supervision. All authors contributed to drafting and review of the manuscript, and approved the submitted version.

## Footnotes

1
http://www.slicer.org

2
https://www.r-project.org
