# Supplementary material for: Radiomics-based infarct features on CT predict hemorrhagic transformation in patients with acute ischemic stroke
Source: Front Neurosci. 2022 Sep 21;16:1002717. doi: 10.3389/fnins.2022.1002717 (PMC9533555; doi:10.3389/fnins.2022.1002717)
Supplement: Supplementary file 2 [file Table_1.docx]

**Table1** The consistency analysis of the diagnosis of two radiologists

| **Image analysis** | **Kappa value** | ***P* value** |
| --- | --- | --- |
| Determinable infarct boundary | 0.754 | 0.000 |
| Dense middle cerebral artery sign | 0.797 | 0.000 |
| Massive cerebral stroke  Hemorrhagic transformation (HT) | 0.744  0.913 | 0.000  0.000 |
